# Partial Miscibility and Concentration Distribution of Two-Phase Blends of Crosslinked NBR and PVC

**DOI:** 10.3390/polym15061383

**Published:** 2023-03-10

**Authors:** Yuka Komori, Aoi Taniguchi, Haruhisa Shibata, Shinya Goto, Hiromu Saito

**Affiliations:** 1 Materials Engineering R & D Division, DENSO CORPORATION, Kariya-shi 448-8661, Aichi, Japan; 2 Department of Organic and Polymer Materials Chemistry, Tokyo University of Agriculture and Technology, Koganei-shi 184-8588, Tokyo, Japan

**Keywords:** blend, NBR, PVC, phase diagram, partial miscibility, DMA, TEM-EDS, elemental mapping

## Abstract

We found that the blends of nitrile butadiene rubber (NBR) and polyvinyl chloride (PVC) exhibited lower critical solution temperature (LCST)-type phase behavior in which a single-phase blend tends to phase separate at elevated temperatures when the acrylonitrile content of NBR was 29.0%. The tan *δ* peaks, which originated from the glass transitions of the component polymers measured by dynamic mechanical analysis (DMA), were largely shifted and broader in the blends when the blends were melted in the two-phase region of the LCST-type phase diagram, suggesting that NBR and PVC are partially miscible in the two-phase structure. The TEM-EDS elemental mapping analysis using a dual silicon drift detector revealed that each component polymer existed in the partner polymer-rich phase, and the PVC-rich domains consisted of aggregated small PVC particles the size of several ten nanometers. The partial miscibility of the blends was explained by the lever rule for the concentration distribution in the two-phase region of the LCST-type phase diagram.

## 1. Introduction

A polymer blend is a mixture of two or more dissimilar polymers that creates a new material. Most pairs of dissimilar polymers with high molecular weights are immiscible, and dissimilar polymers are only miscible when there is a favorable specific interaction between them [1,2,3,4]. The blend of nitrile butadiene rubber (NBR) and polyvinyl chloride (PVC) is known as a miscible blend system and is extensively studied because of its applications in terms of its wire, cable insulation, and automotive parts, due to its excellent oil and chemical resistance, weather resistance, processability, and elasticity [5,6,7,8,9,10]. The miscibility of NBR and PVC is controversial. NBR/PVC blends are considered miscible at wide blend compositions when the acrylonitrile (AN) content of NBR is 23–45% due to a single-glass transition [11,12,13], thermally stimulated depolarization current spectra [14], and negative thermodynamic interaction parameter estimated by inverse gas chromatography [8,15]. On the other hand, a two-phase structure has also been observed in the NBR/PVC blends in the AN content within the same range of 23–45% by a TEM observation [16,17,18]. The difference in the miscibility state might depend on the sample preparation method and blend composition. This might be able to be explained by the existence of the phase diagram, but the phase diagram has not yet been obtained in NBR/PVC blends.

The dissimilar components in polymer blends can be estimated from the compositional dependence of the glass transition [19,20]. A single glass transition can be seen at intermediate temperatures between those of the component polymers in miscible polymer blends due to the cooperative segmental motion of the component polymers [21]. On the other hand, two glass transitions can be seen around the glass transition temperatures of the component polymers in immiscible two-phase blends. The shift and broadness of the glass transition can be seen in various immiscible blends, such as the blend of ethylene propylene diene rubber (EPDM)/styrene butadiene rubber (SBR) [22], NBR/poly(styrene-co-acrylonitrile) (SAN) [23], poly(phenylene sulfide) (PPS)/poly(phenylsulfone) (PPSU) [24], isotactic polypropylene (iPP)/poly(cis-butadiene) rubber (PcBR) [25], acrylate rubber (ACM)/hindered phenol compound, and (AO-80)/chlorinated polypropylene (CPP) [26]. The shift and broadness of the glass transition are usually explained by partial miscibility due to mixing at the interface of the two phases of the component polymers. Hence, the loss tangent (tan *δ*), defined as the ratio of the loss modulus E″ to the storage modulus E′, shifts and becomes broader. Since tan *δ* is a measure of dissipated vibration energy, a broad tan *δ* peak is desired for the application of damping materials widely used to control vibration and noise absorbance in a wide temperature region [27,28,29,30]. The shift and broadness of the glass transition temperatures *T_g_*s have also been reported in NBR/PVC blends due to partial miscibility [31,32,33,34]. However, the shift and broadness are usually slight in two-phase NBR/PVC blends [32,33]. Three phases of an unmixed NBR phase, mixed phase, and PVC microcrystallites are suggested in NBR/PVC blends with a 20% AN content of NBR due to the existence of mixing phases at the interface of peak force quantitative nanomechanical mapping based on atomic force microscopy [35]. The existence of three phases, consisting of the mixed phase, was also suggested by cross-polarization/magic angle spinning ^13^C NMR spectroscopy [17]. Usually, partial miscibility is considered to be attributed to the existence of a mixing region at the interface. Transmission electron microscopy (TEM)–energy dispersive X-ray spectroscopy (EDS) elemental analysis is also often considered available for the observation of the phase structure of polymer blends, but the concentration distribution of the phase structure in the NBR/PVC blend was not obtained by TEM-EDS analysis for the chlorine element of PVC using a lithium–silicon detector [16].

Recently, we found that NBR/PVC blends exhibited lower critical solution temperature (LCST)-type phase behavior, in which a single-phase blend tended to phase separately at elevated temperatures when the AN content of NBR was 29.0%. In this article, to clarify the phase structure of the blends obtained by melting at a temperature in the two-phase region of the LCST-type phase diagram, we investigated the glass transition using the DMA measurement. Direct observation of the concentration distribution in the phase structure was carried out by TEM-EDS elemental mapping analysis for the chlorine element of PVC using a dual silicon drift detector (dual SDD), which is more sensitive to the chlorine element than the lithium–silicon detector. The results are discussed using the lever rule for the concentration distribution in the two-phase region of the phase diagram.

## 2. Materials and Methods

### 2.1. Materials

Nitrile butadiene rubber (NBR) was supplied by Zeon Corp., Tokyo, Japan, grade 1043; the acrylonitrile (AN) content was 29.0% and the Mooney viscosity [ML (1 + 4) 100 °C] was 77.5. Polyvinyl chloride (PVC) was supplied by Taiyo Vinyl Corp., Tokyo, Japan, grade TH-1300. NBR and PVC were used without any further purification. For melt-mixing, a stabilizer for PVC and additives for NBR were used. The stabilizer for PVC was supplied by Adeka Corp., Tokyo, Japan, grade ADK STAB SC-308E. A crosslinking agent of sulfur and the vulcanization accelerators of zinc oxide (ZnO) and stearic acid used in this study were of commercial grade. Sulfur was supplied by Hosoi Chemical Industry Co., Ltd., Tokyo, Japan, grade colloidal sulfur. The vulcanization accelerators of ZnO, stearic acid, N-cyclohexyl-2-benzothiazolylsulfenamide (CZ), and tetramethylthiuram disulfide (TT) were supplied by Hakusui Tech Co., Ltd., Osaka, Japan, grade JIS #2; NOF Corp., Tokyo, Japan, supplied stearic acid camellia; Ouchi Shinko Chemical Industrial Co., Ltd., Tokyo, Japan, supplied Nocceler CZ; and Ouchi Shinko Chemical Industrial Co., Ltd., Tokyo, Japan, supplied Nocceler TT, respectively.

### 2.2. Sample Preparation

The blend specimen was prepared using a solution mixing method and a melt mixing method. For the mixing of the solution, NBR and PVC were dissolved in tetrahydrofuran (THF) with a total weight concentration of 10 wt%. The solutions were cast onto a glass plate at 25 °C, and the solvents were evaporated under cooling air at 25 °C. The cast film was further dried under a reduced pressure at 25 °C for 1 day to completely remove any residual solvent. The prepared solvent-cast blend was then used to obtain the phase diagram.

For melt mixing, NBR and PVC were mixed at 150 °C and at a rotor speed of 200 rpm for 10 min in a mixing chamber of a miniature mixing machine (IMC-18D7, Imoto Machinery Co., Ltd., Kyoto, Japan). In the NBR/PVC blend with a weight ratio of 60/40, the weights of NBR and PVC were about 0.901 g and 0.600 g, respectively. To prevent a chemical reaction between NBR and PVC, the stabilizer for PVC was also added during the mixing, and its weight was about 0.013 g. After mixing, the crosslinking agent sulfur and the vulcanization accelerators ZnO, stearic acid, CZ, and TT were added and mixed at 100 °C for 10 min. These weights were 0.018 g, 0.036 g, 0.012 g, 0.018 g, and 0.002 g, respectively. The obtained blend specimen was then compression molded between two metal plates at 150 °C for 30 min using a hot press machine (Imoto Machinery Co., Ltd., Kyoto, Japan) for cross-linking the NBR. The melt-mixed blend thus prepared was used for the DMA measurement, optical microscopic observation, and TEM observation.

### 2.3. Optical Microscopic Observation

The phase structure was observed under an optical microscope (BX53, Olympus Co., Tokyo, Japan) equipped with a CCD camera (DP74, Olympus Co., Tokyo, Japan).

### 2.4. DMA Measurement

Dynamic mechanical analysis (DMA) was carried out using an RSA G2 (TA Instruments Inc., New Castle, DE, USA) in the tensile mode. The temperature was swept from −60 to 150 °C at a heating rate of 5 °C/min and a constant oscillatory frequency of 10 Hz.

### 2.5. TEM Observation

For the transmission electron microscopy (TEM) observation, an ultrathin section of 100–150 nm thickness was cut from the film specimen with a microtome at −100 °C, and the specimen was stained by RuO_4_ under a vacuum at 20 °C. The morphology was observed under a JEM-F200 TEM instrument (JEOL Co., Ltd., Tokyo, Japan) at an acceleration voltage of 200 kV. The distribution of the component polymer in the observed area was characterized by elemental mapping analysis using energy dispersive X-ray spectroscopy (EDS) equipped with a dual silicon drift detector (dual SDD) (EX-24390UBN5T dry SD100WL, JEOL Co., Ltd., Tokyo, Japan) attached in both a parallel and vertical direction to the sample holder of JEM-F200. The acceleration voltage was 200 kV, and the element concentrations in the line analysis were arranged in atm%.

## 3. Results and Discussion

### 3.1. Phase Behavior

**Figure 1:**
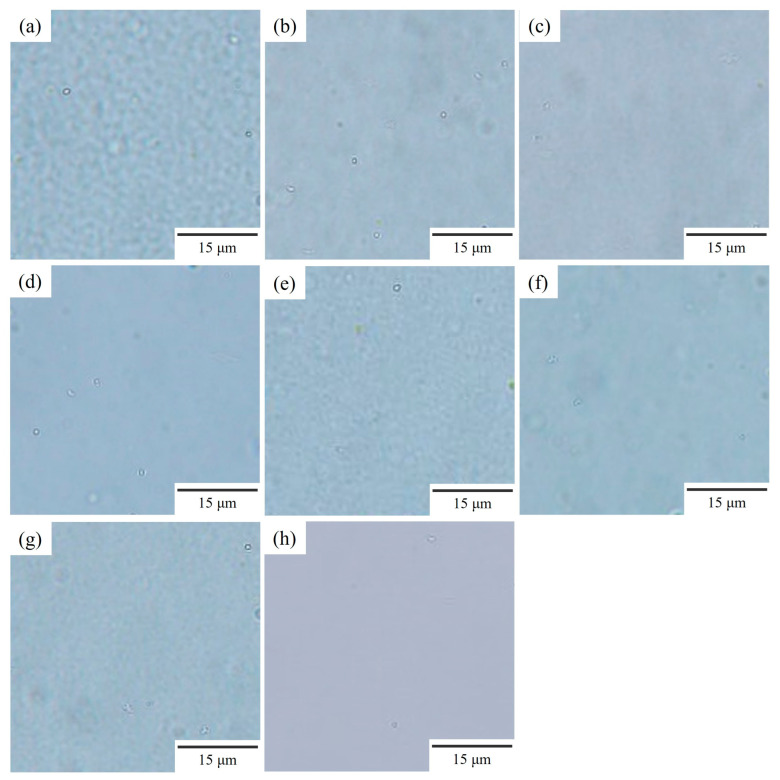
Optical micrographs for phase structure of NBR/PVC blends at various compositions obtained by annealing the solvent-cast films at various temperatures for 1 h: (**a**) 95/5 NBR/PVC, 150 °C; (**b**) 80/20 NBR/PVC, 150 °C; (**c**) 60/40 NBR/PVC, 150 °C; (**d**) 20/80 NBR/PVC, 150 °C; (**e**) 95/5 NBR/PVC, 130 °C; (**f**) 80/20 NBR/PVC, 130 °C; (**g**) 60/40 NBR/PVC, 130 °C; (**h**) 20/80 NBR/PVC, 130 °C.

Figure 1 shows the optical micrographs of the NBR/PVC blends at the various compositions obtained by annealing the solvent-cast films at 130 °C and 150 °C. Here, the acrylonitrile (AN) content of NBR was 29.0%. The solvent-cast film was optically clear, and no structure was seen even after annealing for 1 h at a low temperature below 90 °C. By annealing at a higher temperature, a two-phase structure of a size of around 1 μm was developed by liquid–liquid phase separation, and the film became translucent. The phase-separated structure was seen at a high temperature of 150 °C in NBR/PVC with weight ratios of 95/5, 80/20, 60/40, and 20/80 (Figure 1a–d). Even after annealing for 1 h for the development of phase separation, the contrast of the phase-separated structure was low. The contrast in the structure of the 95/5 NBR/PVC was higher than that of the 80/20, 60/40, and 20/80 NBR/PVC. The high contrast of the structure in the 95/5 NBR/PVC was also observed at a temperature of 140 °C, as shown in Figure S1. Notably, the low-contrast structure shown in Figure 1 could not be observed with our previous CCD camera (Olympus DP73). No structure was seen at the lower temperature of 130 °C in the 20/80 NBR/PVC (Figure 1h), while a phase-separated structure was detected in the 95/5, 80/20, and 60/40 NBR/PVC (Figure 1e–g). These results suggest that NBR/PVC blends exhibit lower critical solution temperature (LCST)-type phase behavior in which a miscible single-phase polymer blend tends to phase-separate at elevated temperatures.

**Figure 2:**
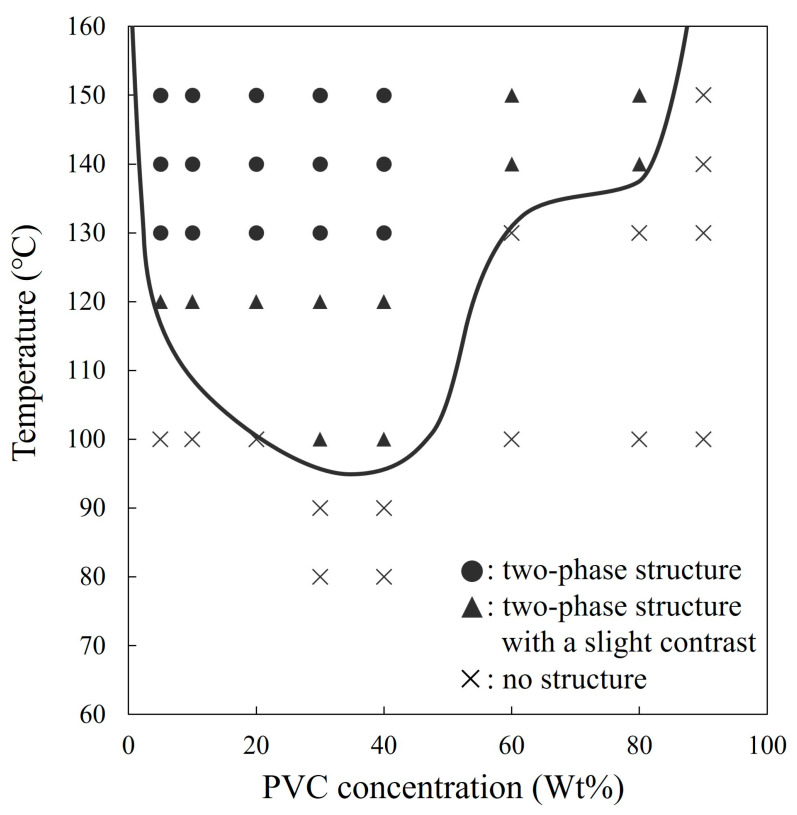
Phase diagram of NBR/PVC blends.

As mentioned above, the solvent-cast films of the NBR/PVC blends were optically clear at all blend compositions, and no structure was detected even after annealing for 1 h at a low temperature below 90 °C. This behavior was observed in the region indicated by crosses in Figure 2. By annealing at a higher temperature, a two-phase structure with a slight contrast, as shown in Figure 1d, was observed in the region indicated by closed triangles in Figure 2. The closed circles in Figure 2 represent the situation in which the two-phase structure with a low contrast was developed by annealing, as shown in Figure 1a–g. On the basis of these observations, an LCST line was drawn, somewhat arbitrarily, in Figure 2. Thus, an LCST-type phase diagram was found to exist in the NBR/PVC blends at an AN content of 29.0%, though the NBR/PVC blends are considered miscible single-phase ones within the AN content range of 23–45%, as reported in the [11,12,13,14,15], while they are considered immiscible two-phase ones in the [16,17,18]. Such a controversial concept might be attributed to the existence of the LCST-type phase diagram, which causes the difference because of the sample preparation method and blend composition. The low contrast of the phase-separated structure shown in Figure 1 is attributed to the phase separation in the two-phase region within the LCST-type phase diagram, as will be described later in Section 3.3. To evaluate the phase diagram exactly, a time-resolved light scattering measurement for the development of the two-phase structure is promising [36,37,38].

**Figure 3:**
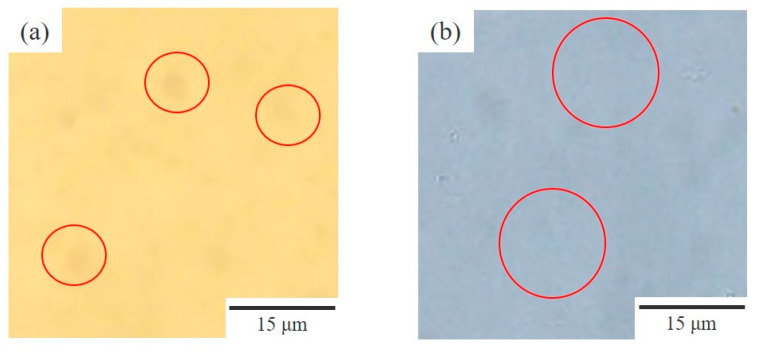
Optical micrographs of 60/40 NBR/PVC: (**a**) melt-mixed blend at 150 °C with additives; (**b**) annealing the solvent-cast film at 150 °C without additives.

Figure 3 depicts the phase structure of the 60/40 NBR/PVC obtained by the melt-mixing and crosslinking of NBR in the two-phase region of 150 °C, which was higher than the LCST line in Figure 2. Here, the NBR in the blend was crosslinked using sulfur with the addition of ZnO and stearic acid as vulcanization accelerators. The phase-separated structure obtained by annealing the solvent-cast blend without additives is also shown in order to make a comparison. Large domains with a size of around 10 μm were dispersed at a small number density in the matrix in the melt-mixed blend (Figure 3a). On the other hand, a small phase structure with a size of around 1 μm obtained by liquid–liquid phase separation was seen at a large number density in the annealed blend without additives (Figure 3b). The size and number density of the domain structure of the melt-mixed blend with additives seen in Figure 3a are quite different from those of the annealed blend without additives (Figure 3b). These results suggest that the large domain structure of the melt-mixed blend shown in Figure 3a is not attributed to the liquid–liquid phase separation of NBR and PVC obtained at the temperature above the LCST phase boundary but to additives such as sulfur and the vulcanization accelerator. That is, the phase-separated structure could not be observed in the melt-mixed blends by optical microscopy due to the low contrast of the phase-separated structure and the low transparency due to the existence of additives, such as sulfur and the vulcanization accelerator. The detail of the phase-separated structure of the melt-mixed blend will be discussed in Section 3.3 by the results of the TEM-EDS elemental mapping analysis using a dual SDD.

### 3.2. Partial Miscibility

**Figure 4:**
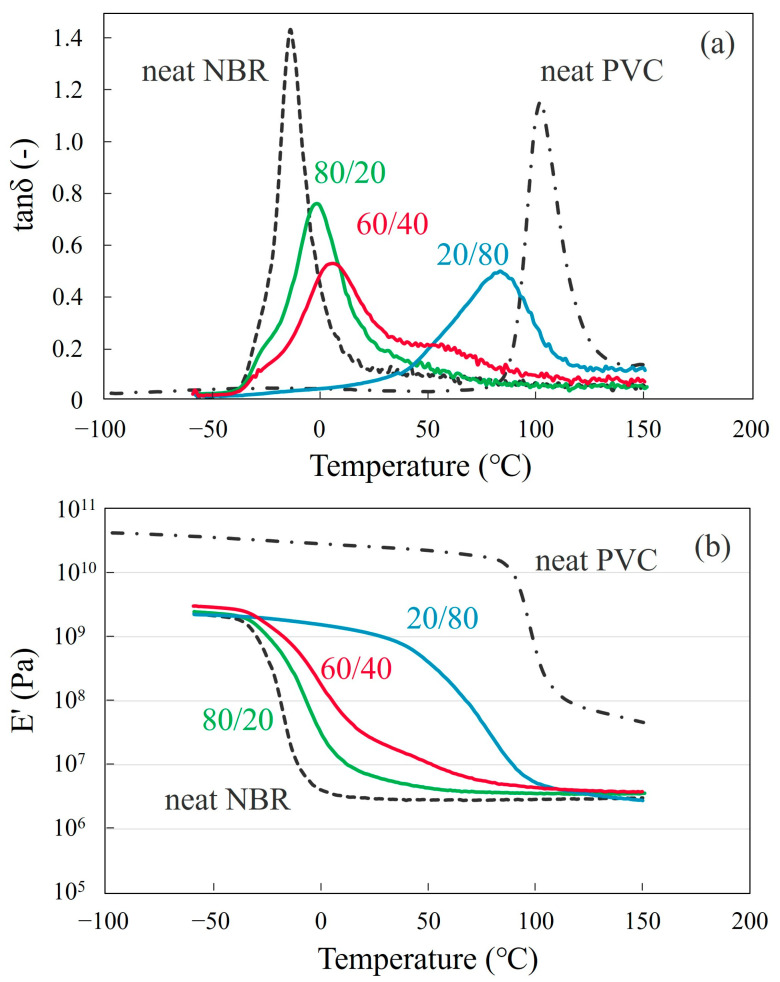
Dynamic mechanical analysis thermograms at a heating rate of 5 °C/min for the melt-mixed NBR/PVC blends at various compositions and the neat component polymers: (**a**) tan δ; (**b**) storage modulus E′.

Figure 4 shows the loss tangent (tan *δ*) and storage modulus E′ with the temperature of the NBR/PVC blends at various compositions and the neat component polymers obtained by the DMA measurements. Here, the blend specimen was prepared by melt-mixing and crosslinking at 150 °C, which was higher than the LCST shown in Figure 2. Tan *δ* peaks derived from the glass transition of neat NBR and neat PVC were observed at around −14 °C and 100 °C, respectively. The 60/40 NBR/PVC blend exhibited two separate tan *δ* peaks (Figure 4a), though a phase structure due to liquid–liquid phase separation could not be observed by optical microscopy (Figure 3a). One peak emerged at around 6 °C, which derived from the glass transition of the NBR phase, and it then shifted to 20 °C higher; the peak width was wider than neat NBR. Another peak emerged at around 56 °C, which derived from the glass transition of the PVC phase, and it then shifted to 44 °C lower; the peak width was wider than neat PVC. That is, large shifts in the glass transitions of the component polymers were seen, and the tan *δ* peaks were broad in the wide temperature region. Owing to the existence of two glass transitions in the 60/40 blend, a gradual two-step change was seen in the E′ (Figure 4b). Since melt-mixing was carried out in the two-phase region of the phase diagram, the shift and width of the glass transitions in the blend suggest the partial miscibility of NBR and PVC in the two-phase structure. As shown in Figure S2, a broad glass transition could not be observed by the DSC measurement, e.g., the glass transition from the NBR-rich phase at around 6 °C could not be observed by the DSC measurement.

On the other hand, a single tan *δ* peak was seen in the 80/20 and 20/80 NBR/PVC blends, though the blends were melt-mixed in the two-phase region in the LCST phase diagram (Figure 4a). The peak position shifted and the peak width was wider in the blends than those of the neat component polymers. The single-glass transition and wide-glass transition regions in the blends were confirmed by the continuous change in the E′ with the temperature (Figure 4b). Though a single-glass transition was seen in the E′, the tan *δ* peak was unsymmetric, and a wide tail was seen in the high-temperature region of 25–80 °C in the 80/20 blend and in the low-temperature region of −10–40 °C in the 20/80 blend. A wide tail was seen in the 80/20 and 20/80 blends due to the small amounts of PVC and NBR in the blends, respectively. Since melt-mixing was carried out in the two-phase region of the phase diagram, an unsymmetric peak with a wide tail and peak shift might be attributed to the partial miscibility of NBR and PVC in the two-phase blends.

**Figure 5:**
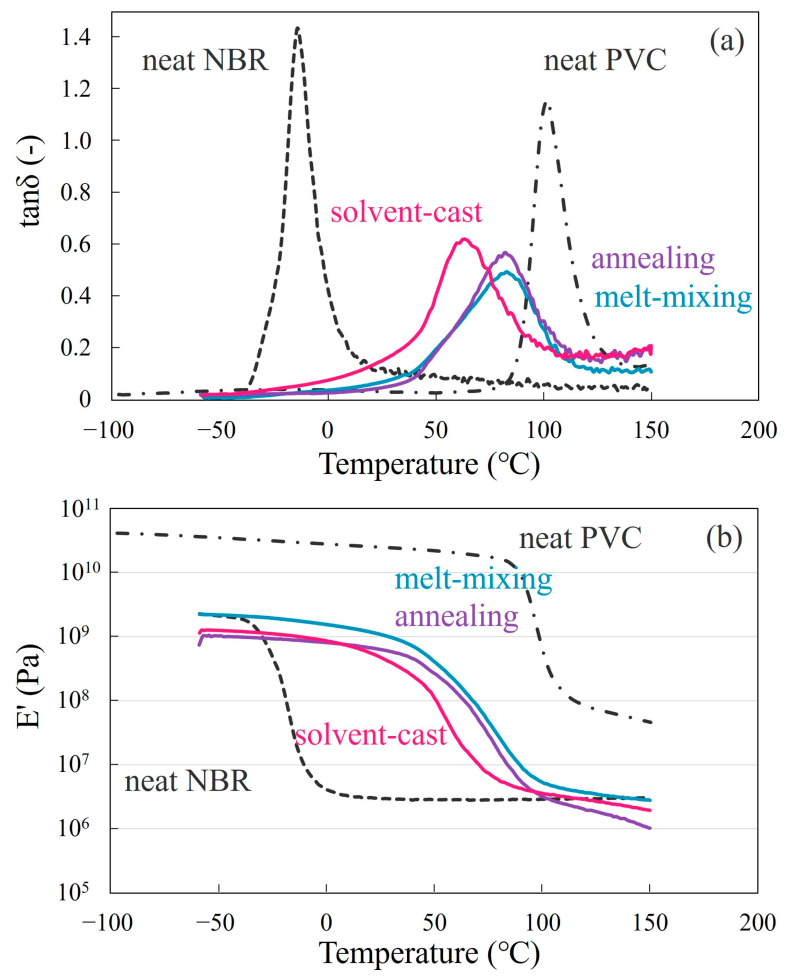
Dynamic mechanical analysis thermograms at a heating rate of 5 °C/min for 20/80 NBR/PVC obtained by solvent-cast, annealing at 150 °C after solvent-cast, and melt-mixing at 150 °C: (**a**) tan δ; (**b**) storage modulus E′.

Figure 5 shows the tan *δ* and storage modulus E′ with the temperature of the 20/80 NBR/PVC blend obtained by the solvent-cast, annealing at 150 °C after the solvent-cast, and melt-mixing at 150 °C. Note here that the tan *δ* and E′ of the 80/20 and 60/40 NBR/PVC blends could not be measured without the crosslinking of the NBR. A single-glass transition was seen in three different specimens. The shape and peak position in the tan *δ* of the annealed blend were almost the same as those of the melt-mixed blend, suggesting that the phase-separated structure of the annealed blend and melt-mixed one are almost the same (Figure 5a). By combining the results of Figure 1d, Figure 4a and Figure 5a, the annealed blend and the melt-mixed one obtained at 150 °C are partially miscible in the two-phase structure. On the other hand, a broad, symmetric peak was seen in the single-phase mixture of the solvent-cast blend. The broader glass transition at a lower glass transition temperature in the solvent-cast blend was confirmed by a gradual change in the wider temperature range and the shift to a lower temperature in the E′ (Figure 5b). Thus, the broad symmetric peak observed at a low temperature in the solvent-cast blend is attributed to the high degree of miscibility, while the broad unsymmetric peak in the annealed blend and melt-mixed one is attributed to the partial miscibility of NBR and PVC in the two-phase structure.

### 3.3. Two-Phase Morphology and Concentration Distribution

**Figure 6:**
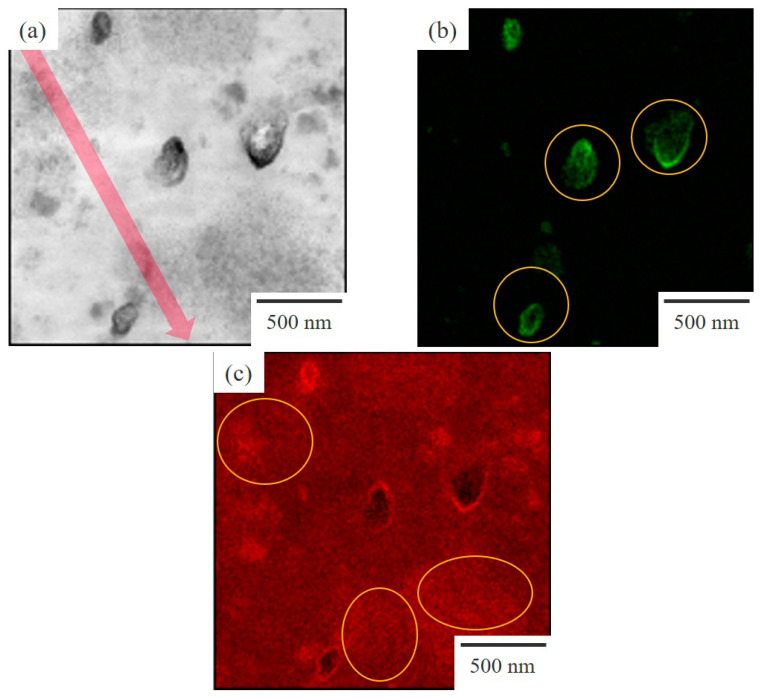
TEM micrographs of 60/40 NBR/PVC obtained by melt-mixing: (**a**) bright field TEM image; (**b**) EDS elemental mapping of zinc; (**c**) EDS elemental mapping of chlorine.

Figure 6 shows a series of TEM micrographs of the 60/40 NBR/PVC prepared by melt-mixing at 150 °C, which was the same for the optical microscopic observation in Figure 3a and the DMA analysis in Figure 4. Here, the thickness of the specimen was 100–150 nm, which was thinner than the phase structure with a size of around 1 μm obtained by liquid–liquid phase separation. Ellipsoidal-shaped domains, with high contrast and a size of about 500 nm, and distorted-shaped domains, which have a size of around several hundred nanometers to several micrometers and consist of the aggregates of small particles with several ten nanometers, were seen in the TEM micrograph stained with RuO_4_ (Figure 6a). To identify these structures, the TEM micrographs observed by energy dispersive X-ray spectroscopy (EDS) elemental mapping analysis using a dual silicon drift detector (dual SDD) with zinc and chlorine as the target elements are shown in Figure 6b,c, respectively. Ellipsoidal-shaped domains with a size of about 500 nm were seen in the TEM-EDS mapping image of the zinc target, indicating that the ellipsoidal-shaped domains with strong contrast, as shown in Figure 6a, are assigned to the vulcanization accelerator of ZnO (Figure 6b). The ZnO domains with a size of about 500 nm were much smaller than those of the large domains with a size of around 10 μm shown in Figure 3a, suggesting that the large domains shown in Figure 3a are not assigned to ZnO but instead to the crosslinker sulfur. On the other hand, small particles of several ten nanometers were seen in the TEM-EDS mapping image of the chlorine target, indicating that the distorted-shaped domains consisting of the aggregates of small particles were assigned to the PVC phase (Figure 6c). The interesting result here is that chlorine was also detected in the matrix area outside of the PVC phase, indicating that PVC exists in the matrix outside of the PVC domains.

**Figure 7:**
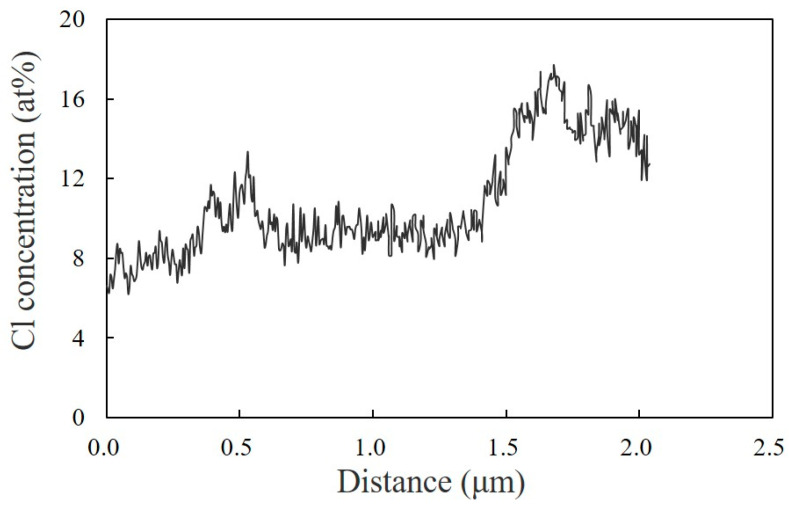
Concentration distribution in chlorine of PVC for the melt-mixed 60/40 NBR/PVC in the region indicated by a red line in Figure 6a.

Owing to the high sensitivity of the chlorine element by TEM-EDS elemental mapping analysis using a dual SDD, a distribution in the concentration of the chlorine of PVC in the melt-mixed 60/40 NBR/PVC could be obtained, as shown in Figure 7. Here, the concentration distribution was obtained in the region indicated by a red line in Figure 6a. The concentration of chlorine was higher in the domain and lower in the matrix, yet it was not 0 in the matrix. The distorted-shaped domains consisting of the aggregates of small particles were assigned to the PVC-rich phase, while the matrix outside of the distorted domains was assigned to the NBR-rich one. This indicates that each component polymer exists in the partner polymer-rich phase, i.e., PVC exists in the NBR-rich matrix and NBR exists in the PVC-rich domain. Thus, the partial miscibility suggested by the DMA measurement shown in Figure 4 and Figure 5 is attributed to the existence of PVC in the NBR-rich matrix and that of NBR in the PVC-rich domain. This concept is different from the partial miscibility caused by mixing at the interphase in the two-phase structure. Owing to the existence of each of the component polymers in the partner polymer-rich phase, the contrast of the two-phase structure observed by the optical microscope is low, as shown in Figure 1 and Figure 3.

**Figure 8:**
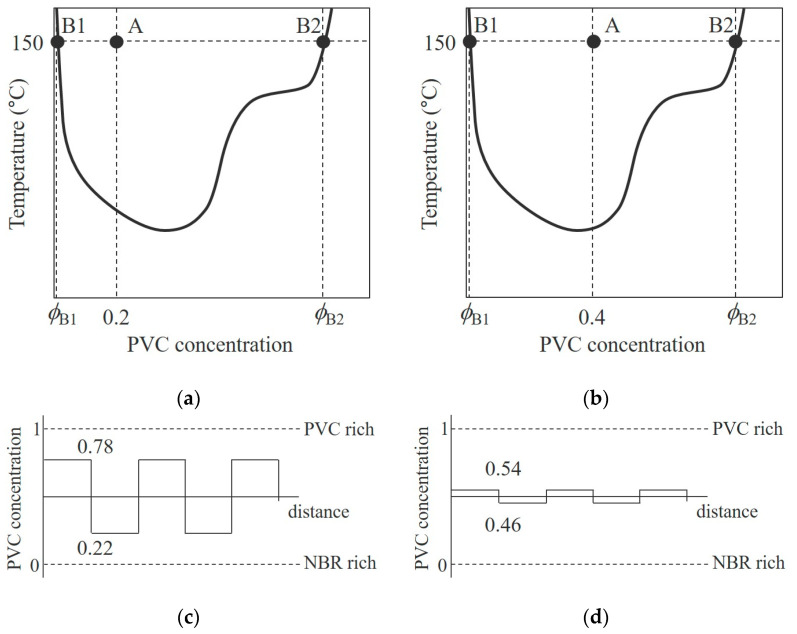
Schematic illustrations of phase diagram and concentration distribution in the NBR/PVC blends: (**a**,**c**) *ϕ*_A_ = 0.2, (**b**,**d**) *ϕ*_A_ = 0.4.

Figure 8 shows schematic illustrations for the LCST-type phase diagram obtained from Figure 2 and the corresponding concentration distribution in the NBR/PVC blends estimated by the lever rule when NBR and PVC are phase-separated in the two-phase region within the LCST-phase diagram at different blend compositions. When the blend is phase-separated at point A in Figure 8a,b, for the blend of the PVC concentration of *ϕ*_A_, NBR and PVC never phase-separate into their pure components; instead, they phase-separate into NBR-rich and PVC-rich phases. This indicates that each of the component polymers exists in the partner polymer-rich phase, as suggested by the TEM-EDS elemental mapping analysis shown in Figure 6 and Figure 7. According to the lever rule, the concentrations of the PVC component in the NBR-rich phase *f*_NBR_ and that in the PVC-rich phase *f*_PVC_ are given by:

(1) $$f_{NBR}=\frac{\phi_{A}-\phi_{B1}}{\phi_{B2}-\phi_{B1}}$$

(2) $$f_{PVC}=\frac{\phi_{B2}-\phi_{A}}{\phi_{B2}-\phi_{B1}}$$

where *ϕ*_B1_ and *ϕ*_B2_ are the PVC concentrations at points B1 and B2 in the phase diagram of Figure 8, respectively. When the blend of *ϕ*_A_ = 0.2 is phase-separated at 150 °C (Figure 8a), the concentrations of PVC in each rich phase are *f*_NBR_ = 0.22 and *f*_PVC_ = 0.78 (Figure 8c). On the other hand, when the blend of *ϕ*_A_ = 0.4 is phase-separated at 150 °C (Figure 8b), the concentrations of PVC in each rich phase are *f*_NBR_ = 0.46 and *f*_PVC_ = 0.54 (Figure 8d).

Thus, the concentration difference in the *f*_NBR_ and *f*_PVC_ |*f*_NBR_ − *f*_PVC_| estimated in the 60/40 NBR/PVC was smaller than that in the 80/20 NBR/PVC. Owing to the small concentration difference |*f*_NBR_−*f*_PVC_| in the NBR/PVC blends, the contrast of the phase-separated structure was slight or low, as shown in Figure 1 and Figure 3. Thus, the partial miscibility suggested by the large shift in the glass transitions of the component polymers estimated by the DMA measurements shown in Figure 4 and the low contrast of the phase-separated structure shown in Figure 1 is attributed to the phase separation in the two-phase region of the LCST-type phase diagram. This concept is different from the partial miscibility caused by mixing at the interface of the two-phase structure.

## 4. Conclusions

We found that the blend of NBR/PVC exhibited an LCST-type phase diagram when the AN content of NBR was 29.0%. The glass transition temperatures *T_g_*s of NBR and PVC measured by DMA were largely shifted and broader in the blends after melting in the two-phase region of the LCST-type phase diagram. The *T_g_* of NBR was shifted from −14 °C to 6 °C, and that of PVC was shifted from 100 °C to 56 °C in the 60/40 NBR/PVC, suggesting that NBR and PVC are partially miscible in the two-phase structure. A morphological observation by TEM-EDS elemental mapping analysis using dual SDD revealed that each of the component polymers existed in the phase of the partner polymer, and the PVC-rich phase consisted of distorted domains aggregated by small PVC particles the size of several ten nanometers. The partial miscibility of the blend is attributed to the liquid–liquid phase separation in the two-phase region of the LCST-type phase diagram. Owing to the partial miscibility due to the liquid–liquid phase separation, largely shifted tan *δ* peaks were observed in the wide temperature region. This concept is helpful for controlling tan *δ* by blending dissimilar polymers for damping materials.

## Data Availability

Not applicable.

## Supplementary Materials

The following supporting information can be downloaded at: https://www.mdpi.com/article/10.3390/polym15061383/s1, Figure S1: Optical micrographs for phase structure of NBR/PVC blends at various compositions obtained by annealing the solvent-cast films at 140 °C for 1 h: (a) 95/5 NBR/PVC; (b) 80/20 NBR/PVC; (c) 60/40 NBR/PVC; (d) 20/80 NBR/PVC; Figure S2: DSC thermograms at a heating rate of 10 °C/min for the melt-mixed NBR/PVC blends at various compositions and the neat component polymers.
