# Supplementary material for: Partial Miscibility and Concentration Distribution of Two-Phase Blends of Crosslinked NBR and PVC
Source: Polymers (Basel). 2023 Mar 10;15(6):1383. doi: 10.3390/polym15061383 (PMC10058492; doi:10.3390/polym15061383)
Supplement: Supplementary file 1 [file polymers-15-01383-s001.zip › polymers-2241122-supplementary.pptx]

## Slide 1
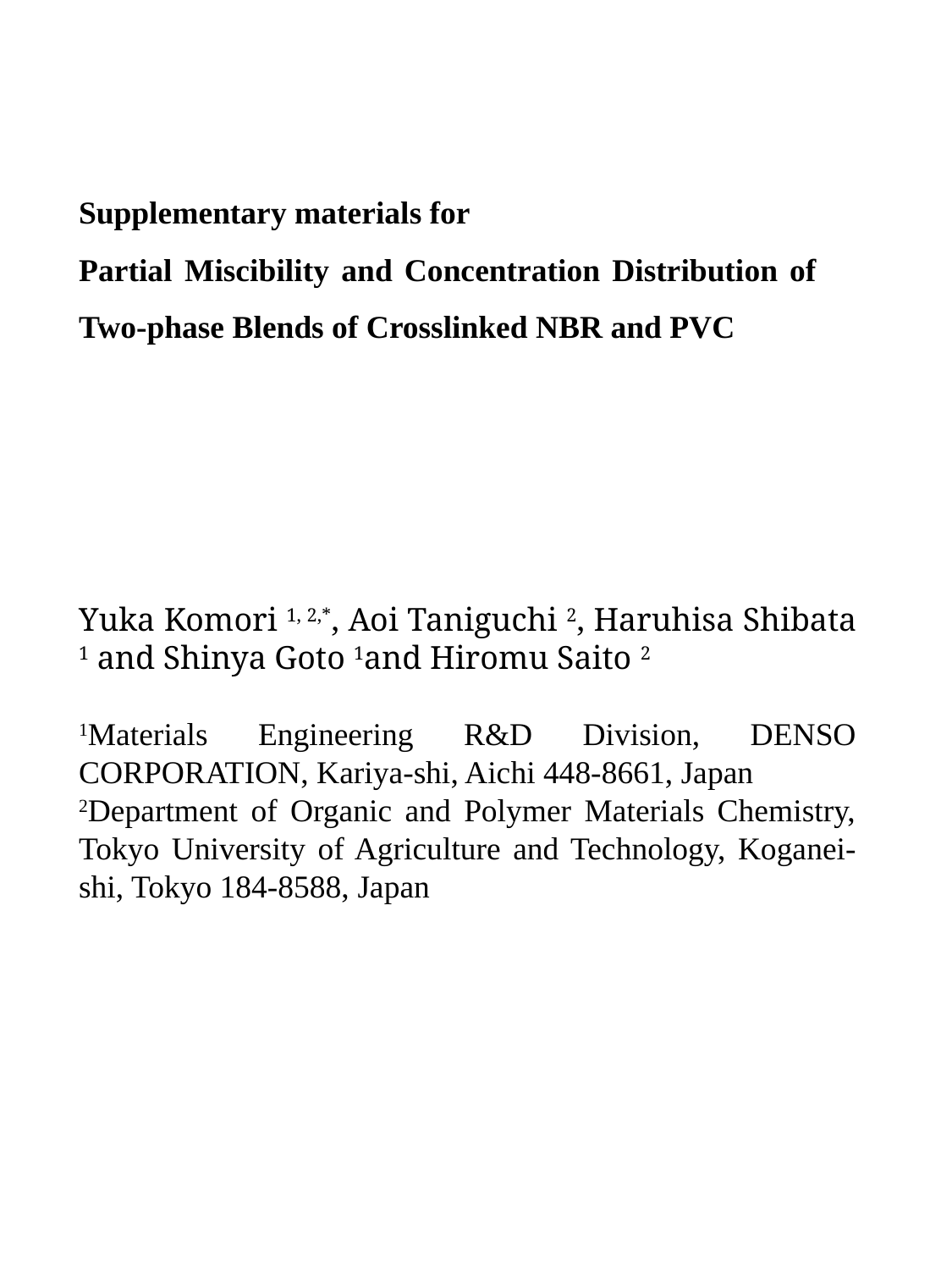

Supplementary materials for
Partial Miscibility and Concentration Distribution of Two-phase Blends of Crosslinked NBR and PVC
Yuka Komori 1, 2,*, Aoi Taniguchi 2, Haruhisa Shibata 1 and Shinya Goto 1and Hiromu Saito 2
1Materials Engineering R&D Division, DENSO CORPORATION, Kariya-shi, Aichi 448-8661, Japan
2Department of Organic and Polymer Materials Chemistry, Tokyo University of Agriculture and Technology, Koganei-shi, Tokyo 184-8588, Japan

## Slide 2
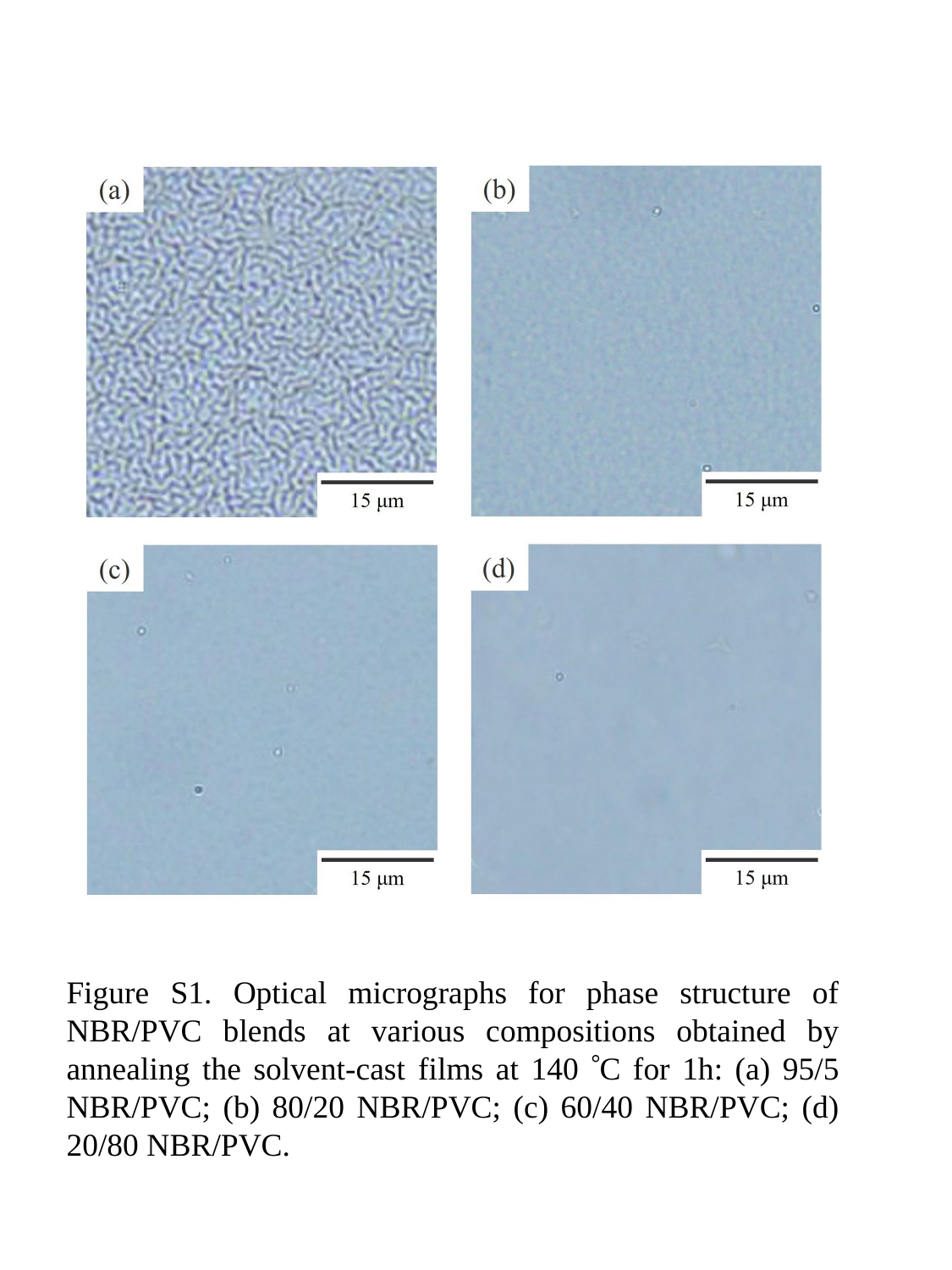

Figure S1. Optical micrographs for phase structure of NBR/PVC blends at various compositions obtained by annealing the solvent-cast films at 140 °C for 1h: (a) 95/5 NBR/PVC; (b) 80/20 NBR/PVC; (c) 60/40 NBR/PVC; (d) 20/80 NBR/PVC.

## Slide 3
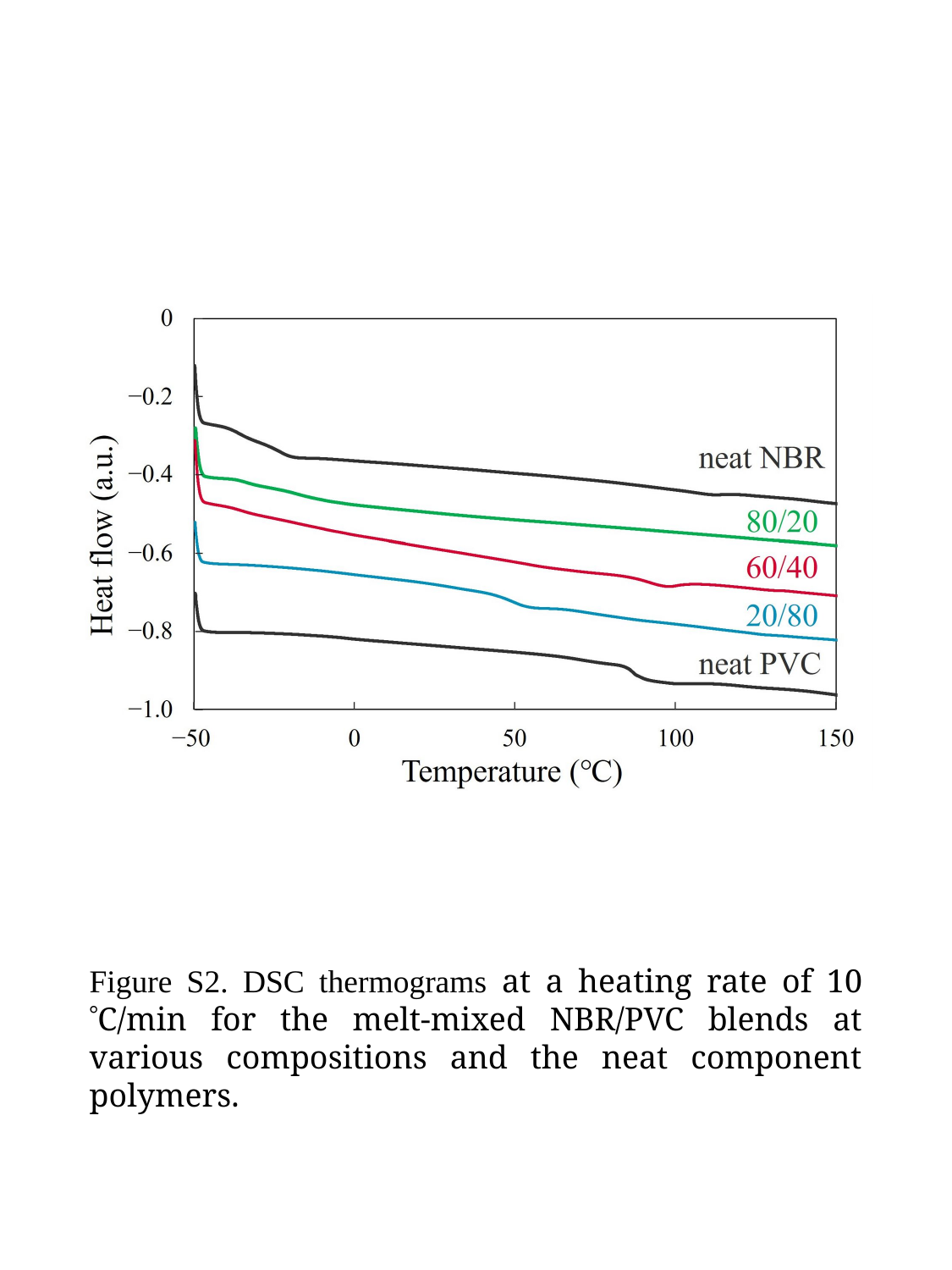

Figure S2. DSC thermograms at a heating rate of 10 °C/min for the melt-mixed NBR/PVC blends at various compositions and the neat component polymers.
